# *Porphyromonas gingivalis* and Its Outer Membrane Vesicles Induce Neuroinflammation in Mice Through Distinct Mechanisms

**DOI:** 10.1002/iid3.70135

**Published:** 2025-02-11

**Authors:** Yu Qiu, Yueyang Zhao, Guiqiong He, Deqin Yang

**Affiliations:** ^1^ Chongqing Key Laboratory of Oral Diseases Chongqing Municipal Key Laboratory of Oral Biomedical Engineering of Higher Education, Stomatological Hospital of Chongqing Medical University Chongqing China; ^2^ Department of Conservative Dentistry and Endodontics Shanghai Stomatological Hospital & School of Stomatology Fudan University Shanghai China; ^3^ Center for Neuroscience Research Chongqing Medical University Chongqing China; ^4^ Advanced Innovation Center for Human Brain Protection, Beijing Tiantan Hospital Capital Medical University Beijing China

**Keywords:** blood‒brain barrier, neuroinflammation, NLRP3 inflammasome, outer membrane vesicles, *Porphyromonas gingivalis*, systemic inflammation

## Abstract

**Background:**

Alzheimer's disease (AD) is the most common chronic neurodegenerative disorder, with neuroinflammation playing an important role in its progression to become a major research focus. The role of *Porphyromonas gingivalis* (*Pg*) and its outer membrane vesicles (*Pg* OMVs) in AD development is uncertain, particularly regarding their effects on neuroinflammation.

**Methods:**

The cognition of mice injected with *Pg*, *Pg* OMVs, or PBS via the tail vein was assessed by the Morris water maze test. Pathological changes in the mouse brain were analyzed via immunohistochemistry, immunofluorescence and hematoxylin‒eosin (H&E) staining, and the ultrastructure of the hippocampus was observed via transmission electron microscopy (TEM). Plasma levels of inflammatory factors were assessed by enzyme‐linked immunosorbent assay (ELISA). Protein levels of brain inflammatory factor, occludin, and NLRP3 inflammasome‐related proteins were assessed by western blotting.

**Results:**

Memory impairment; notable neuroinflammation, including astrocyte and microglial activation; and elevated protein levels of IL‐1β, TNF‐α, and IL‐6 in the hippocampus were detected in the *Pg* and *Pg* OMV groups. However, *Pg* induced weight loss and systemic inflammation, such as splenomegaly and increased IL‐1β and TNF‐α levels in plasma, whereas *Pg* OMVs had minimal impact. In addition, *Pg* induced more pronounced activation of the NLRP3 inflammasome compared to *Pg* OMVs. In contrast, only the *Pg* OMV group exhibited blood−brain barrier (BBB) disruption characterized by reduced integrity of tight junctions and lower levels of occludin protein.

**Conclusions:**

*Pg* is associated with a significant immune response and systemic inflammation, which in turn exacerbates neuroinflammation via activating NLRP3 inflammasome. However, *Pg* OMVs might elude the systemic immune response and disrupt tight junctions, thereby entering the brain and directly triggering neuroinflammation.

## Graphical abstract

Pg is associated with a significant immune response and systemic inflammation, which in turn exacerbates neuroinflammation and cognitive dysfunction. However, Pg OMVs might elude the systemic immune response and disrupt tight junctions, thereby entering the brain and directly triggering neuroinflammation. Our findings not only uncover distinct pathways through which Pg and Pg OMVs contribute to disease progression but also further confirm Pg OMVs as important regulators of Pg‐induced AD‐like pathological changes.

**Abbreviations**
AD: Alzheimer's disease
BBB: blood−brain barrier
BCA: bicinchoninic acid
CNS: central nervous system
ELISA: enzyme‐linked immunosorbent assay
H&E: hematoxylin‒eosin
IHC: immunohistochemistry
LPS: lipopolysaccharide
NLRP3: NOD‐like receptor protein 3
OMVs: outer membrane vesicles
*Pg*
: *Porphyromonas gingivalis*
*Pg* OMVs: *Porphyromonas gingivalis* outer membrane vesicles
TEM: transmission electron microscopy
WB: western blotting

## 1 Introduction

Alzheimer's disease (AD) is a prominent neurodegenerative disease worldwide characterized by progressive memory deficiency, behavioral changes, and impaired self‐care abilities [1, 2]. Recent epidemiological and experimental evidence supports a hypothesis for infection in which pathogens, for example, bacteria, viruses, and prions, may be common causative agents for the onset of AD [3]. *Porphyromonas gingivalis* (Pg), a Gram‐negative rod bacterium and the main anaerobic pathogen involved in the development of periodontitis, is also considered a factor contributing to AD pathogenesis [4]. Indeed, a recent one bidirectional Mendelian randomization study suggested that a positive association exists between *Pg* infection and the development of AD [5]. Virulence factors produced by *Pg*, including lipopolysaccharide (LPS), gingipains, and *Pg* DNA, have been found in the brain tissues of AD patients [6, 7]. According to many experimental studies, *Pg* or its virulence factors may cause cognitive dysfunction and AD‐like pathological changes [8, 9, 10]. However, as one of the body's highest oxygen tension environments, the brain is not ideal for *Pg* growth. As a direct link has yet to be established for the presence of *Pg* in AD brains [11], the exact mechanism and role of the microbe in AD pathogenesis remain uncertain.

Outer membrane vesicles (OMVs) are nanoparticles surrounded by a bilayer of proteolipids [12] that enclose a variety of bacterial components, such as microbe‐associated molecular patterns (MAMPs) and virulence factors to damage normal host tissues [13, 14]. In biomedical fields, OMVs have gained substantial attention because of their intrinsic role in delivering bacterial cargo to distal recipients [15]. Interestingly, in mice colonized by pathogenic *Escherichia coli* (*E. coli*), OMVs disseminate extensively to the heart, liver, kidney, spleen, and brain [16], leading to the assumption that *Pg* OMVs detected in mouse brains in our previous study could be one etiology of memory impairment and AD in vivo [17]. However, other studies suggest that *Pg* OMVs administered via oral gavage are undetected in hippocampal tissue and thus do not contribute to cognitive impairment [18]. Therefore, the effect of *Pg* OMVs on cognition remains controversial and requires further validation.

Cognitive impairment among AD patients is closely associated with neuroinflammation [19], a defense mechanism that initially protects the brain through pathogen clearance or inhibition [20]. However, this inflammatory response has dual effects on the host, with both beneficial effects in promoting tissue repair and eliminating metabolic waste with synchronous persistent inflammatory stimuli that are detrimental to the host [21]. Infection or inflammatory diseases culminate in the release of proinflammatory factors from peripheral organs into the blood resulting in systemic inflammation and neuroinflammation [22]. Periodontitis compromises systemic health primarily through the spread of subgingival plaque biofilm bacteria to extraoral tissues, causing bacteremia [23, 24]. Bacteremia associated with periodontitis may play a major role in causing systemic inflammation and neuroinflammation. As the major pathogen of periodontitis, *Pg* has been proposed to impact AD. Nevertheless, the potential virulence effects of *Pg* and *Pg* OMVs on systemic inflammation and neuroinflammation are less well understood [25]. Therefore, we injected *Pg* and *Pg* OMVs separately via the tail vein to establish a mouse model of bacteremia to investigate their effects on systemic inflammation and neuroinflammation. We found that *Pg* and *Pg* OMVs induce cognitive deficits by promoting neuroinflammation in mice. Moreover, we identified the neuroinflammation induced both indirectly and directly by *Pg* and *Pg* OMVs, which trigger systemic inflammation and disrupt the blood‒brain barrier (BBB), respectively. This study provides new insights into the role of *Pg* and *Pg* OMVs in the onset and development of AD.

## 2 Materials and Methods

### 2.1 *P. gingivalis* Culture and OMV Isolation

*Pg* ATCC 33277 was provided by West China Hospital of Stomatology, Sichuan University. *Pg* were cultured in brain heart infusion broth medium and collected after 48 h of growth in an anaerobic chamber (80% N_2_, 10% O_2_, 10% H_2_) at 37°C. The culture medium was subsequently centrifuged to separate the bacteria from the supernatant. The *Pg* OMV isolation protocol followed a previously described procedure [26]. Then, the *Pg* OMVs were washed with phosphate‐buffered saline (PBS) and centrifuged again. After being dissolved in 200 μL of PBS, the samples were refrigerated at ultralow temperatures until use. A bicinchoninic acid (BCA) protein assay kit (Beyotime, China) was used to detect the protein concentration of the OMVs. The phenotype, concentration, and size distribution of *Pg* OMV particles were measured via Zeta View (Particle Metrix, Germany).

### 2.2 Animals

All experiments complied with the National Research Council's Guide for the Care and Use of Laboratory Animals and were approved by the Institutional Animal Care and Use Committee of Chongqing Medical University (NO. IACUC‐CQMU‐2023‐0160). A total of 40 10‐month‐old male C57BL/6J mice weighing 25−30 g were obtained from and housed in the Animal Center of Chongqing Medical University. Food and water were freely available to the animals at approximately 22°C under specific‐pathogen‐free (SPF) conditions. The *Pg* and *Pg* OMV bacteremia model was established following previous procedures [27]. The animals were randomized to each group (*n* = 10/group). For unbiased results, a computer‐generated random number table was used along with blinding procedures. In this study, we found that 10^9^ CFU of *Pg* could produce *Pg* OMVs containing approximately 100 μg of protein. The groups were injected intravenously with 100 μL of PBS (control group, Con), 10^7^ CFU of *Pg* in 100 μL of PBS (*Pg* group, *Pg*), or 1 μg of *Pg* OMVs in 100 μL of PBS (*Pg* OMVs group, OMV) through the tail vein three times a week for 12 weeks. The body weights of the mice were recorded every week. At the end of the study, the mice were euthanized, and the weights of the spleens were recorded immediately after spleen removal.

### 2.3 Morris Water Maze Test

After a day of acclimatization, each mouse was placed in a cylindrical pool 120 cm in diameter, and the mice subsequently searched for a visible circular platform (10 cm in diameter) that was 1 cm above the surface of the water for 120 s from each quadrant in turn. If the mice failed to find the platform during the 2 min search, they were guided to the target for 20 s until they could find it. The next day, the platform was hidden 1 cm below the surface of the water so that the mice could not see it. For the 4‐day hidden platform trial, the test procedure was the same as that in the visible platform phase. In the last phase, the platform was dismantled. Each mouse was placed diagonally opposite the platform quadrant and allowed to search for the platform site for 120 s. The swimming trail and the learning and motion parameters of the mice were recorded with a video tracking system (Bio‐Rad). Data analysis was completed with the support software Smart v3.0.

### 2.4 Transmission Electron Microscopy (TEM)

TEM is widely used for analyzing the size, shape, and ultrastructure of various biological components, including OMVs. Freshly extracted OMVs were diluted 3000 times onto a copper mesh grid with 2% phosphotungstic acid and dried. Anesthetized mice were perfused with 20 mL each of ice‐cold PBS and 4% glutaraldehyde. The hippocampus were separated and cut into 1 mm^3^ blocks. Postfixations in 1% osmium tetroxide and graded ethanol were performed after the samples were fixed in 4% glutaraldehyde for 12 h. The samples were then photographed via TEM (Hitachi, Tokyo, Japan).

### 2.5 Immunohistochemistry (IHC) and Hematoxylin‒Eosin (H&E) Staining

After being embedded in paraffin, the brain tissues were cut into 5 μm thick slices. The samples were dewaxed and rehydrated prior to blocking endogenous peroxidase activity with 3% H_2_O_2_. After being washed with PBS, the samples were blocked in 5% bovine serum albumin (Beyotime, China) for 30 min and then incubated with the following primary antibodies: rabbit anti‐GFAP (1:50, #80788, CST, USA) and rabbit anti‐Iba1 (1:200, DF6442, Affinity Biosciences, USA) overnight at 4°C. After being washed with PBS, the sections were incubated for 1 h with a secondary antibody. After a 5 min incubation with diaminobenzidine (Beyotime, China), the sections were counterstained with hematoxylin (Beyotime, China). Finally, the sections were observed via optical microscopy (Olympus Corporation, Japan). GFAP‐ and Iba1‐positive cells in the sections were analyzed with ImageJ (NIH ImageJ system, USA). H&E staining was performed according to the manufacturer's instructions (Solarbio Science & Technology, China), and the samples were viewed under an optical microscope (Olympus Optical Co. Ltd., Tokyo, Japan).

### 2.6 Enzyme‐Linked Immunosorbent Assay (ELISA)

Blood samples from the mice were collected by eye removal and placed in tubes containing ethylenediaminetetraacetic acid (EDTA). Following centrifugation at 3000 rpm for 5 min at 4°C, the plasma was collected, and the levels of interleukin (IL)‐10 (#88‐7105), IL‐1β (#88‐7013), and TNF‐α (#88‐7324) were measured via ELISA kits (Invitrogen, USA).

### 2.7 Western Blotting (WB)

After the mice were humanely killed, mouse hippocampal tissues were dissected and lysed with RIPA buffer supplemented with PMSF and phosphatase inhibitors (Beyotime, China). Equal amounts of total protein (30−60 μg) were separated by SDS‒PAGE and transferred to nitrocellulose (NC) membranes (Millipore, USA) via a Trans‐Blot Turbo system (Bio‐Rad). The sections were incubated with primary antibodies after antigen blocking. The primary antibodies used were as follows: Gingipain (61BG1.3, 1:300, Developmental Studies Hybridoma Bank, USA), TNF‐α (#11948, 1:1000, CST, USA), IL‐1β (#31202, 1:1000, CST, USA), IL‐6 (#DF6087, 1:1000, Affinity Biosciences, USA), Occludin (13409‐1‐AP, 1:2000, Proteintech, USA), NLRP3 (19771‐1‐AP, 1:1000, Proteintech, USA), ASC/TMS1 (#67824, 1:1000, CST, USA), Caspase 1 (#AF5418, 1:1000, Affinity Biosciences, USA), Cleaved‐Caspase 1 (#AF4022, 1:1000, Affinity Biosciences, USA), and GAPDH (5174S, 1:1000, CST, USA) overnight at 4°C. The NC membranes were subsequently incubated with a secondary antibody for 60 min after washing. Blotted bands were detected with an Odyssey Clx (LI‐COR, USA) or Bio‐Rad imaging system (Bio‐Rad, Hercules, USA) and analyzed with ImageJ software (NIH ImageJ system, USA).

### 2.8 Immunofluorescence (IF)

The brain sections were incubated with rabbit anti‐NLRP3 (19771‐1‐AP, 1:100, Proteintech, USA), rabbit anti‐CD31 (28083‐1‐AP,1:200, Proteintech, USA), and mouse anti‐Occludin (sc‐133256, 1:50, Santa Cruz, USA) at 4°C overnight. The sections were then incubated with the corresponding secondary antibodies at room temperature for 1.5 h: CoraLite488‐conjugated Goat Anti‐Rabbit (1:200, SA00013‐2, Proteintech) for binding to NLRP3; Cy3, Goat Anti‐Rabbit (1:200, E031620‐02, EarthOx), and CoraLite488‐conjugated Goat Anti‐Mouse (1:200, SA00013‐1, Proteintech) were utilized to bind to CD31 and occluding, respectively. Finally, the sections were sealed using the anti‐fluorescence fading mounting medium containing DAPI (S2110, Solarbio). Fluorescent signals were detected using confocal microscopy. The fluorescence intensity was analyzed using ImageJ software.

### 2.9 Statistical Analysis

Statistical analyses were performed via GraphPad Prism 9, and the data are presented as the means ± standard deviations (SDs). Unless stated otherwise, at least three independent experiments were conducted for each experiment. We chose the sample size based on past experiences with experimental variability. Unpaired two‐tailed Student's *t‐*test and one‐way analysis of variance followed by post hoc Tukey's multiple comparisons were used to compare the differences between two groups and three groups, respectively, for normally distributed data. For non‐normally distributed data, the Mann−Whitney *U* test and Kruskal−Wallis test with Dunn's multiple comparisons test were used to compare the differences between two groups and more than two groups, respectively. Statistical significance was defined as *p* < 0.05.

## 3 Results

### 3.1 Characterization of *Pg* and *Pg* OMVs

**Figure 1:**
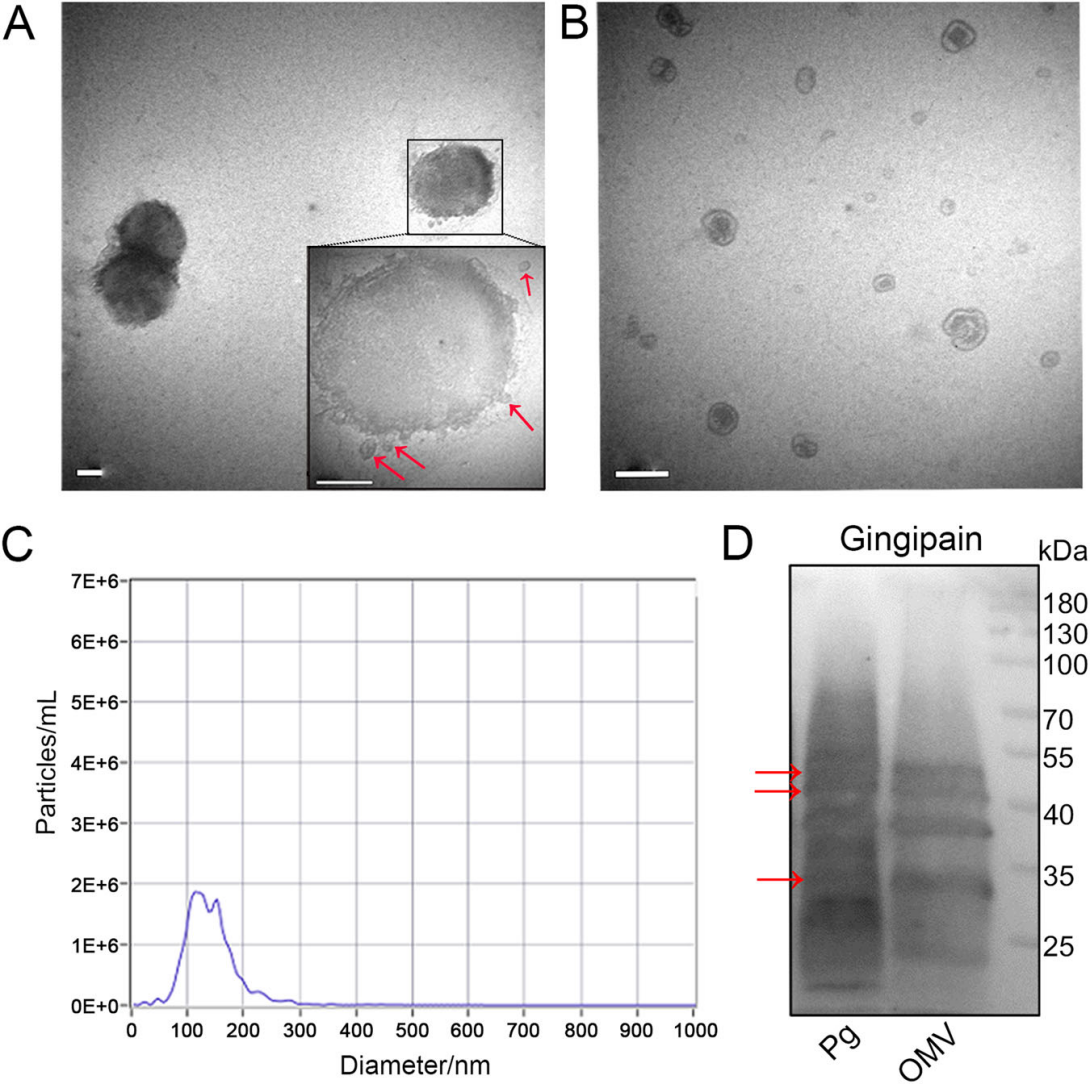
*Pg* and its OMV characterization. (A) The *Pg* outer membrane produces numerous OMVs. The red arrows indicate the OMVs secreted from *Pg*. (B) The purified vesicles from the *Pg* culture supernatant show the bilayer single membrane surrounded by nanosized spherical particles. Scale bars = 200 nm. (C) Distribution of the particle sizes of *Pg* OMVs. (D) Immunoblots of *Pg* OMVs and *Pg* proteins (61BG1.3).

*Pg* and its OMVs were photographed via TEM. The secretion of OMVs from *Pg* was observed, and subsequent blebbing was monitored (red arrows; Figure 1A). The *Pg* OMV size distribution was tested via nanoparticle tracking analysis, with OMV diameters ranging between 50 and 280 nm in diameter (Figure 1B,C). *Pg* OMVs were further confirmed by enrichment of gingipain (*P. gingivalis* hemagglutinin), the major virulence factor of *Pg* that is also present in OMVs, with multiple bands consistent with *Pg* (Figure 1D).

### 3.2 *Pg* and *Pg* OMVs Induce Spatial Reference Memory Impairment in C57BL/6J Mice

**Figure 2:**
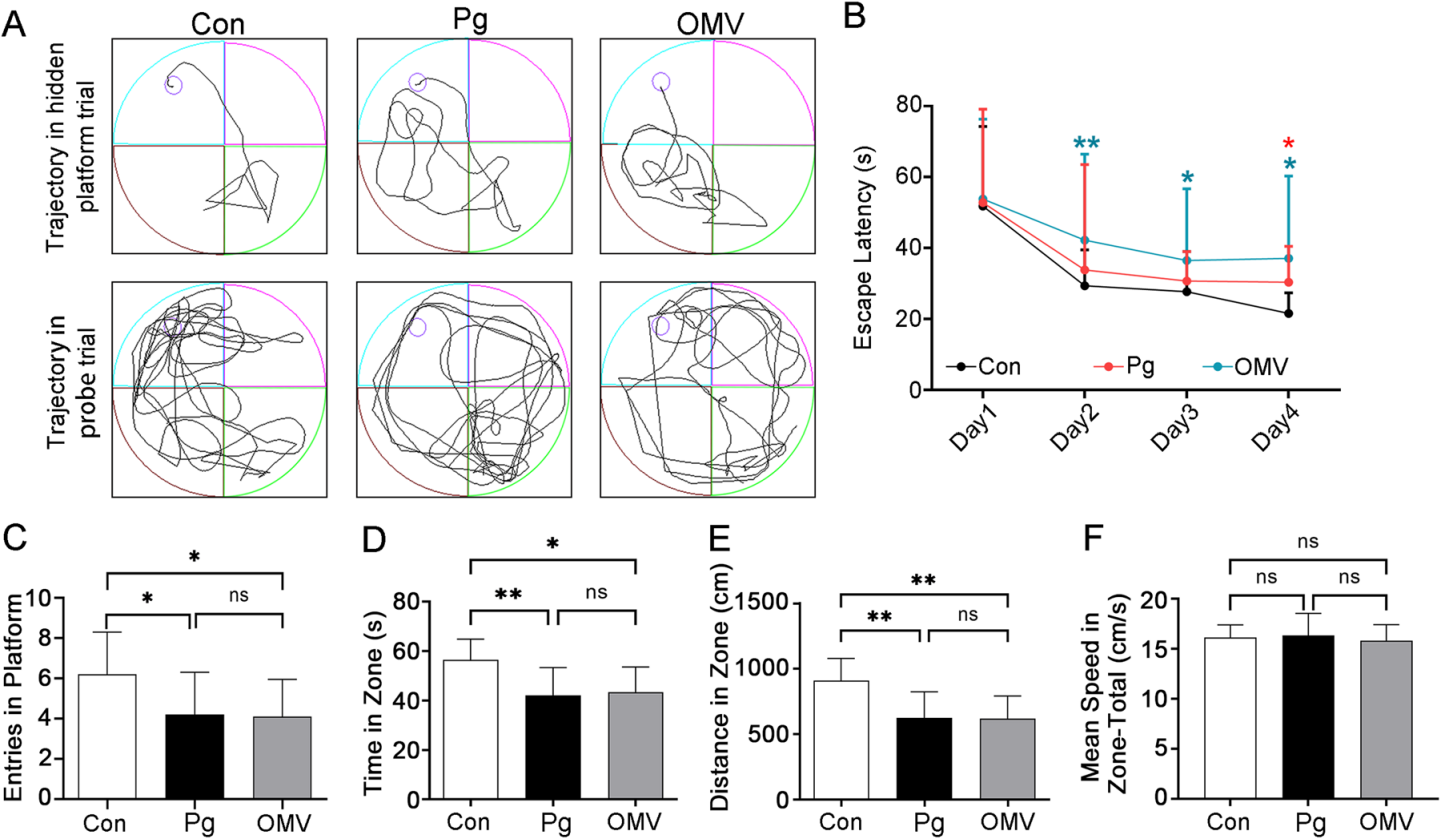
*Pg* and *Pg* OMVs weakened spatial memory in the mice. (A) Representative tracking images of the mice in the hidden platform trial and the probe trial. (B) Escape latency in the target quadrant during the hidden platform trial. (C−E) The number of platform crossings, time spent, and travel distance in the target quadrant during the probe trial. (F) The mean swimming speed in the total area. Con, treated with PBS; *Pg*, treated with *Pg*; OMV, treated with *Pg* OMVs. *n* = 10. *p < 0.05; **p < 0.01; ns: not significant.

First, we investigated the effects of *Pg* and *Pg* OMVs on cognitive function. After 12 weeks of treatment, we examined the effects on spatial reference memory via the Morris water maze test (Figure 2). The representative trials during the hidden platform trial and probe trial of the three groups of mice are shown in Figure 2A. Compared with the Con group, the *Pg*‐treated and *Pg*‐OMV‐treated groups presented longer escape latencies on the fourth day (Figure 2B), fewer platform crossings (Figure 2C) and shorter travel time (Figure 2D) and distances (Figure 2E) in the target quadrant. However, except for an earlier prolonged escape latency in the *Pg*‐OMV group than in the Con group (Figure 2B), *Pg* and *Pg*‐OMV groups did not significantly differ. The three groups of mice had similar mean swimming speeds (Figure 2F), helping to rule out an effect of motor ability on other experimental data. These findings indicate that both *Pg* and *Pg* OMVs lead to cognitive deficits in mice.

### 3.3 *Pg* and *Pg* OMVs Trigger Neuroinflammation in C57BL/6J Mice

**Figure 3:**
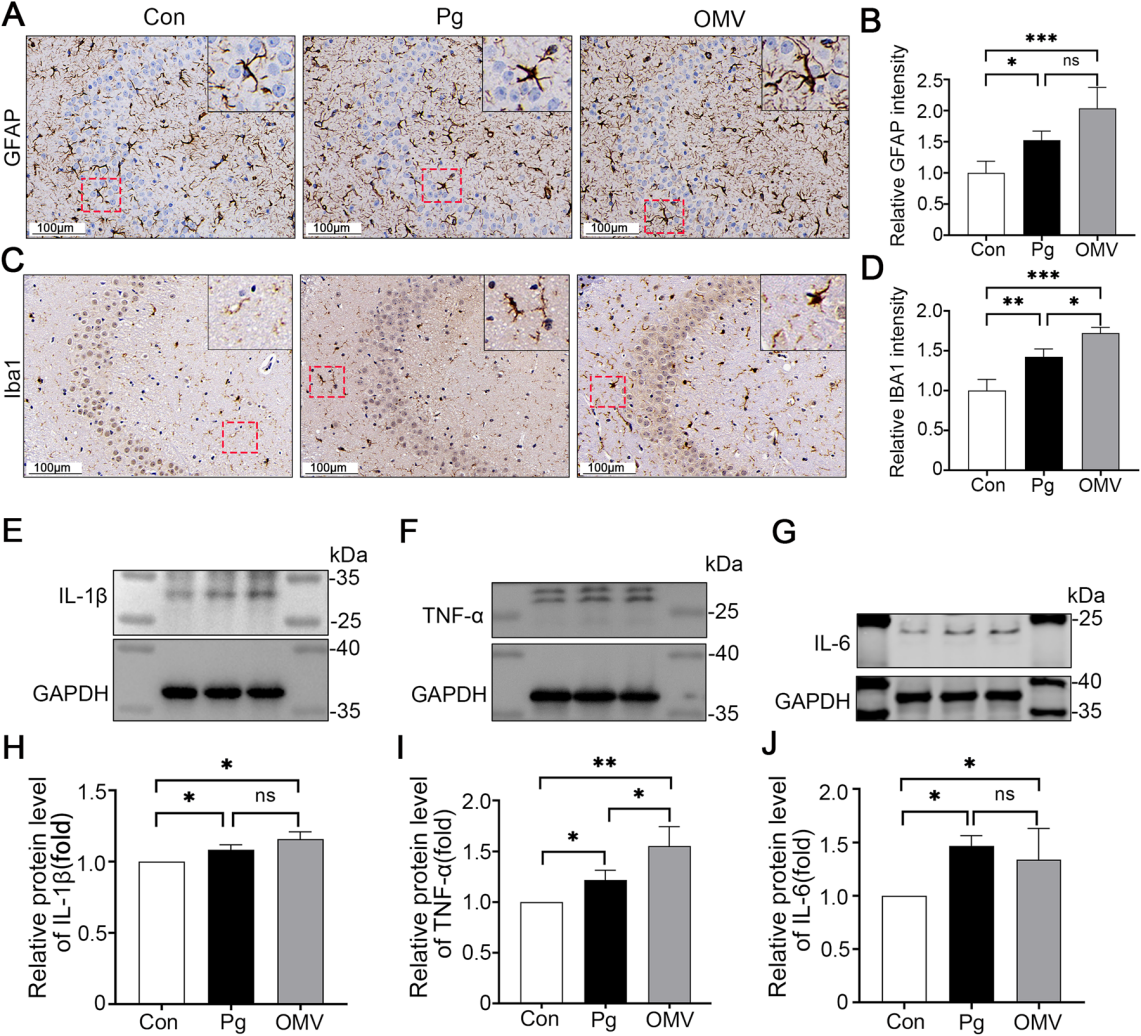
*Pg* and *Pg* OMVs induced neuroinflammation in the hippocampus. (A, C) Immunohistochemistry of GFAP and Iba‐1 in hippocampi from the Con, *Pg,* and *Pg* OMVs groups. Scale bars: 100 μm. (B, D) Quantification of the relative GFAP and Iba1 intensity in the hippocampus. *n* = 4. (E−J) IL‐1β, TNF‐α, and IL‐6 protein expression in the hippocampus. *n* = 3. *p < 0.05; **p < 0.01; *** *p* < 0.001; ns: not significant.

Among neurodegenerative diseases, including AD, neuroinflammation is a significant pathological hallmark [28], and is modulated by astrocytes and microglia that reside in the central nervous system (CNS) [19]. Then, we employed IHC to investigate differences in the activation of GFAP^+^ astrocytes and Iba1^+^ microglia in the mice brains of three groups. Compared to those in the Con group, significant increases in GFAP and Iba1 signals were observed in the hippocampus, which represents the glial activation (Figure 3A−D). The astrocytes and microglia in the cortical regions of both the *Pg* and *Pg* OMVs groups also exhibited comparable levels of activation (Supporting Information S1: Figure S1). Furthermore, after *Pg* OMVs and *Pg* treatment, the protein levels of inflammatory mediators, including interleukin 1β (IL‐1β), tumor necrosis factor‐α (TNF‐α), and IL‐6 in the hippocampus were significantly greater than those in the Con group (Figure 3E−J). Notably, TNF‐α expression was significantly greater in the *Pg* OMVs group than in the *Pg* group (Figure 3F,I).

Taken together, these findings suggest that mice treated with *Pg* and *Pg* OMVs exhibit neuroinflammation, which contributes to the development of cognitive deficits.

### 3.4 *Pg* Causes Severe Systemic Inflammation in C57BL/6J Mice

**Figure 4:**
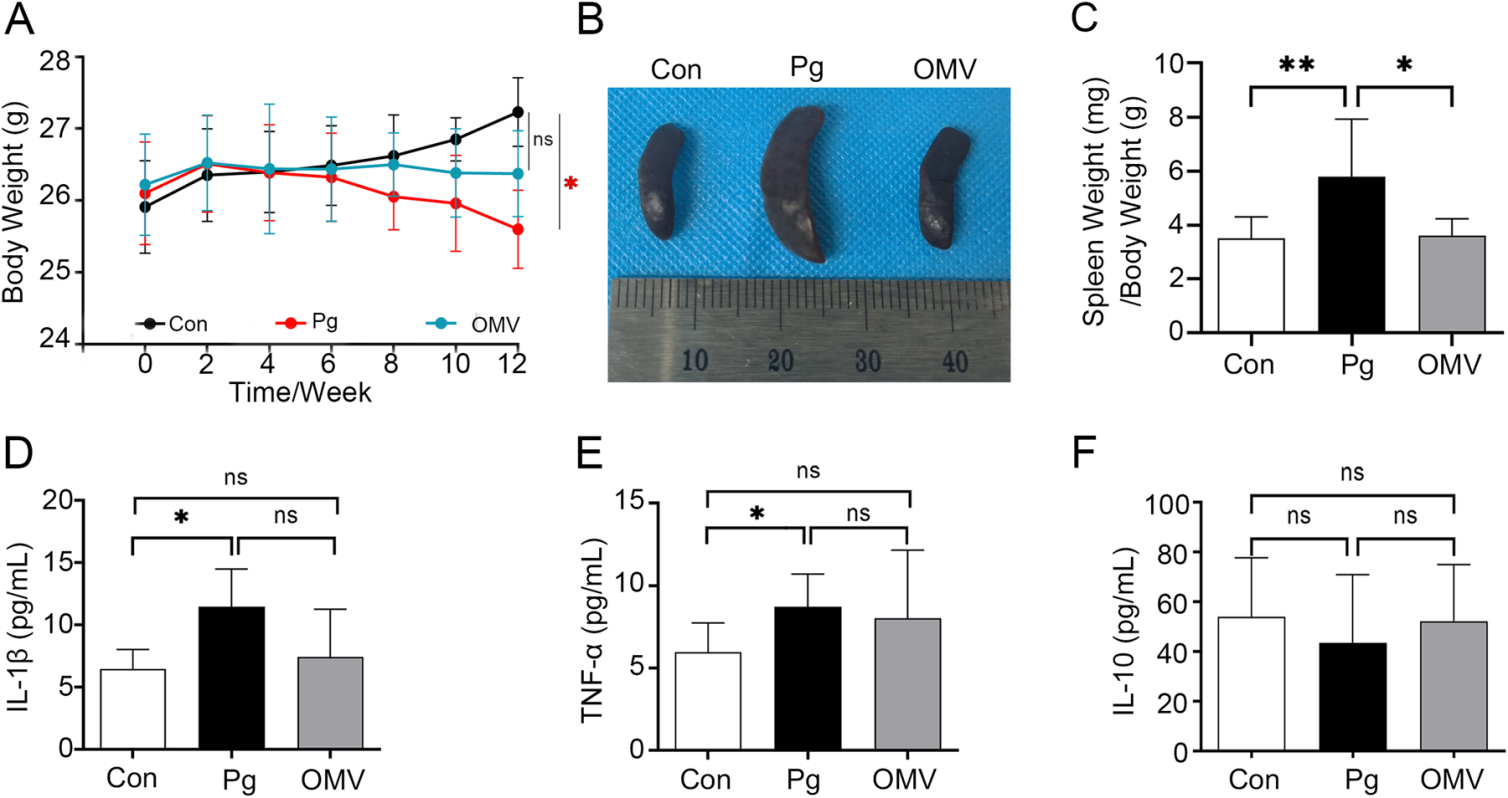
*Pg* induced systemic inflammation in mice. (A) Effects on body weight after intravenous injection of *Pg*, *Pg* OMVs, or PBS via the tail. (B) Representative image of the spleen. *n* = 10. (C) Spleen/body weight ratios. (D−F) Inflammatory cytokine concentrations in plasma (IL‐1β, TNF‐α, and IL‐10). *n* = 6. *p < 0.05; **p < 0.01; ns: not significant.

The effects of systemic peripheral inflammation have been demonstrated to induce neuroinflammation [29], which prompted us to assess changes in body weight, ratios between the wet weight of the spleen (mg) and the body weight (g) (spleen index), and levels of plasma inflammatory cytokines after *Pg* and OMVs intravenous tail injection. After *Pg* injection, a reduction in body weight was observed (*p *= 0.0385, Figure 4A), concomitant with an increase in the spleen index (*p *= 0.0079, Figure 4B,C). Furthermore, levels of plasma inflammatory cytokines IL‐1β and TNF‐α were significantly elevated in the *Pg* group compared with the Con group (*p* = 0.0500, *p *= 0.0289; Figure 4D,E). In contrast, following the injection of *Pg* OMVs, the spleen index and plasma concentrations of IL‐1β and TNF‐α were consistent with the corresponding levels in the Con group (Figure 4A−E). However, there were no significant effects on the plasma concentrations of the anti‐inflammatory cytokine IL‐10 in the three groups, although a slight decrease was noted in the *Pg* group (Figure 4F).

Collectively, these findings indicate that *Pg* can initiate systemic inflammation, which in turn induces neuroinflammation.

### 3.5 *Pg* and *Pg* OMVs Differentially Activate the NLRP3 Inflammasome in the Hippocampus of Mice

**Figure 5:**
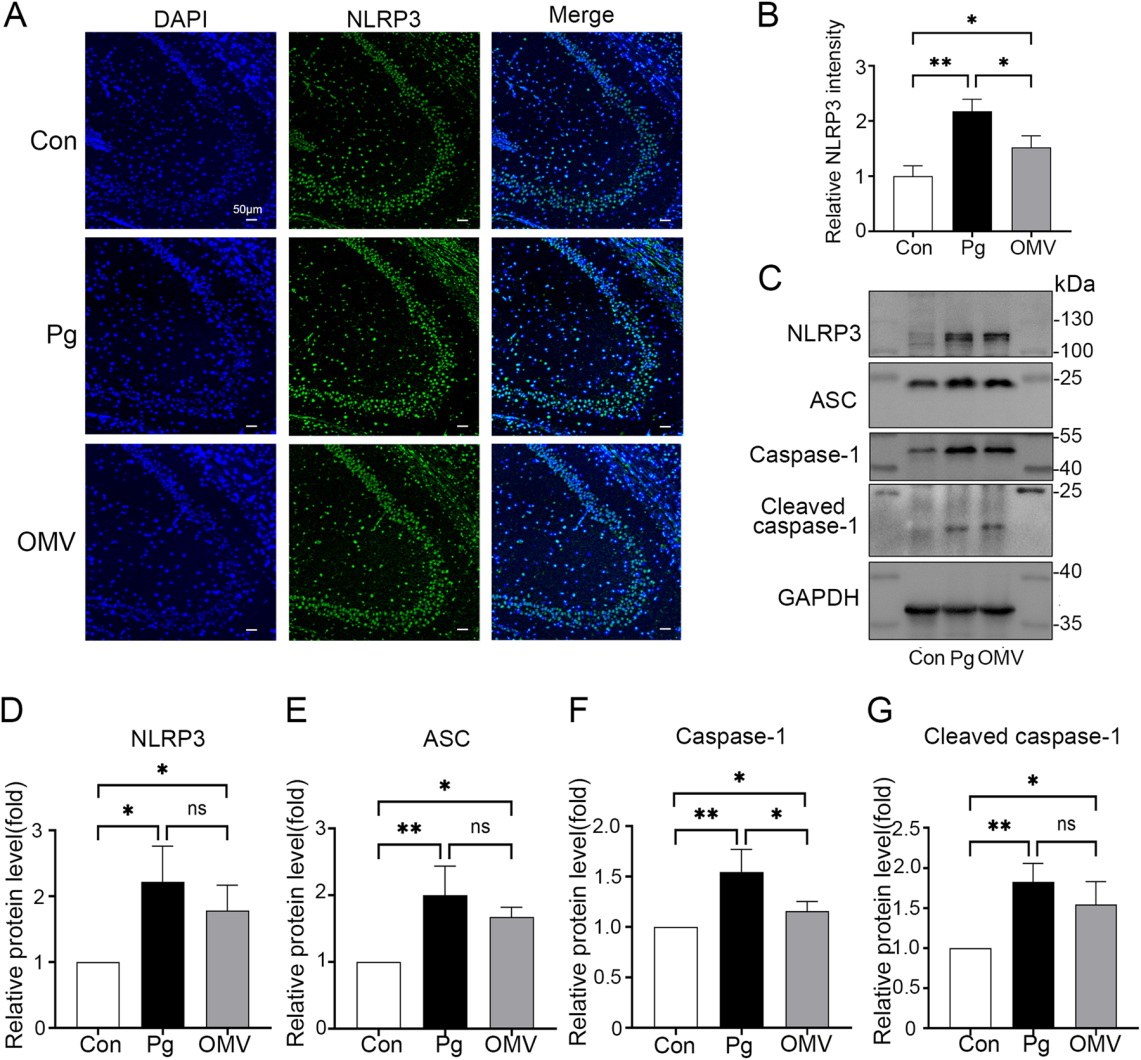
*Pg* and *Pg* OMVs increased NLRP3 inflammasome‐related protein levels in the hippocampus. (A) Immunofluorescence of NLRP3 in hippocampi from the Con, *Pg,* and *Pg* OMVs groups. Scale bars: 50 μm. (B) The quantification of the relative intensity of NLRP3 in the three groups. *n* = 3. (C−F) NLRP3, ASC, Caspase‐1, and Cleaved caspase‐1 protein expression in the hippocampus. *n* = 3. *p < 0.05; **p < 0.01; ns: not significant.

The NOD‐like receptor protein 3 (NLRP3) inflammasome, an inflammatory complex activated in response to various inflammatory or injurious stimuli, plays a crucial role in the pathogenesis of neuroinflammation, with its assembly and activation pivotal to this process [30, 31]. To investigate the specific mechanisms of cognitive deficits in mice induced by *Pg* and *Pg* OMVs, we assessed whether the NLRP3 inflammasome was activated. IF imaging revealed a significant increase in NLRP3 fluorescence intensity in the hippocampus of both the *Pg* and *Pg* OMVs groups (Figure 5A,B). To further explore the effects of *Pg* and *Pg* OMVs on the NLRP3 inflammasome in the hippocampus, the WB assay was used to quantify the proteins of NLRP3, Apoptosis‐associated speck‐like protein containing a caspase recruitment domain (ASC), caspase‐1 and cleaved caspase‐1 expressions. In accordance with the IF results, expressions of these molecules were elevated significantly in the hippocampus of these two groups (Figure 5C−F). Interestingly, both the IF and WB results showed a more pronounced increase in the *Pg* group compared to the *Pg* OMVs group, which may be related to the severe systemic inflammation caused by *Pg*. These results suggest that *Pg* and *Pg* OMVs may induce neuroinflammation by activating the NLRP3 inflammasome to different extents.

### 3.6 *Pg* OMVs Compromise the Integrity of the BBB of C57BL/6J Mice

The alterations in neuroinflammation are closely correlated with the extent of BBB dysfunction [32]. *Pg* and *Pg* OMVs have been reported to have the potential to traverse the BBB and possibly inflict damage on the brain [17, 27]. Thus, we investigated how *Pg* and *Pg* OMVs impact the BBB to understand their distinct roles in cognitive alterations in mice.

**Figure 6:**
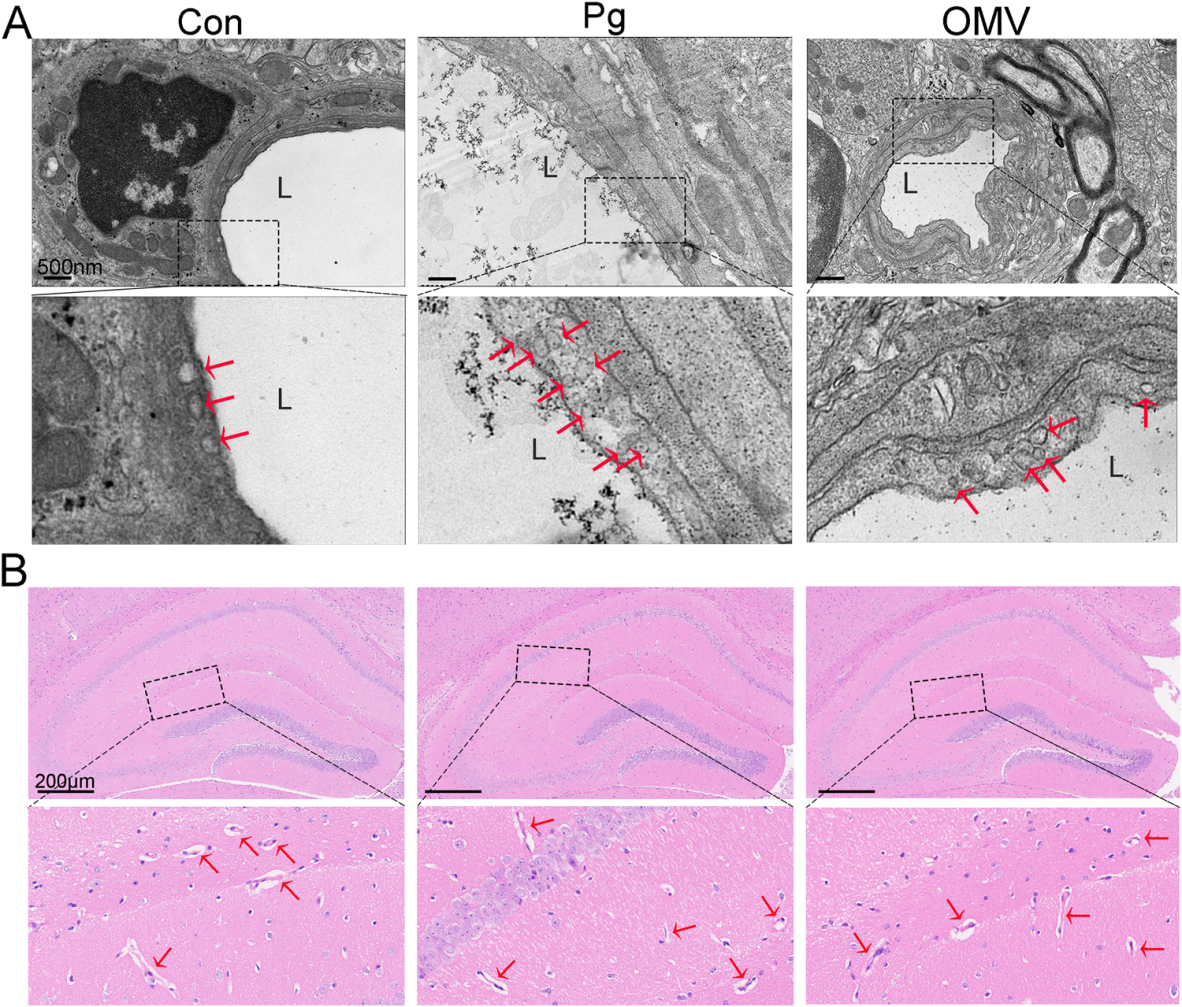
*Pg* and *Pg* OMVs promoted the formation of vesicles in brain endothelial cells but did not cause vascular dilation in the hippocampus. (A) TEM images of the ultrastructures of vesicles in brain endothelial cells (red arrows). Scale bar: 500 nm. (B) Representative HE‐stained images of the hippocampus. Red arrows indicate cerebral vessels. Scale bar: 200 μm. L, lumen.

**Figure 7:**
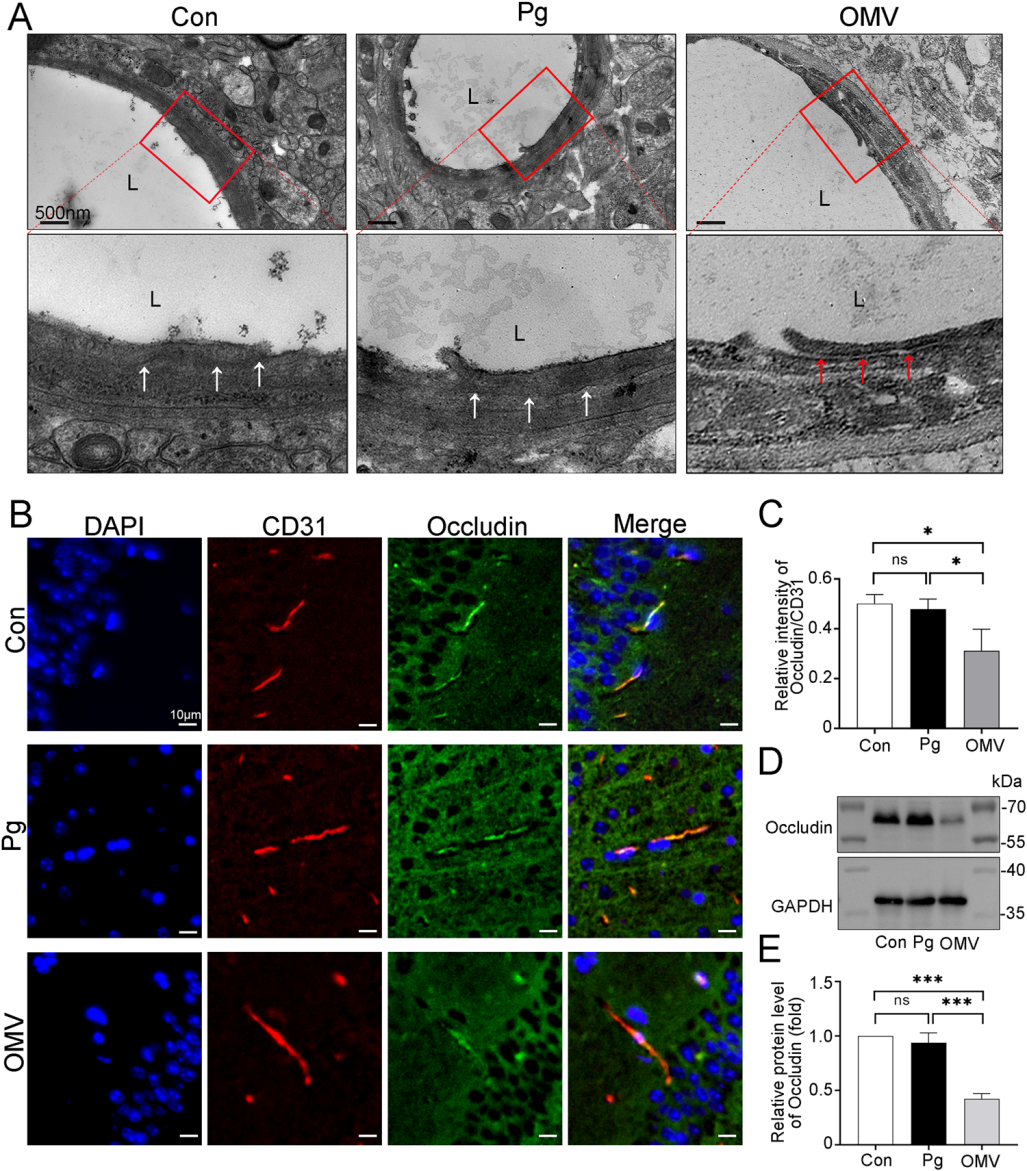
*Pg* OMVs disrupt the integrity of tight junctions in brain endothelial cells. (A) TEM images of the ultrastructures of tight junctions in the hippocampus. The normal tight junctions (white arrows) with high electron density and abnormal tight junctions (red arrows) with low electron density are similar to a gap. Scale bar: 500 nm. (B) Representative images of double IF staining of CD31 and occludin in the hippocampus. Scale bar: 10 μm (C) The ratio of Occludin^+^ to CD31^+^ positive staining areas. *n* = 4. (D, E) Occludin protein levels in the hippocampus. *n* = 3. *p < 0.05; *** *p* < 0.001, ns: not significant. L, lumen.

The TEM images revealed an increased number of vesicles in the endothelial cells of the hippocampus in the *Pg* and *Pg* OMVs groups compared with those in the Con group (Figure 6A), which was consistent with the results reported in the literature [27]. However, no significant dilation of blood vessels has been observed in the brains of the three groups of mice so far (Figure 6B, Supporting Information S1: Figure S2). Moreover, no *Pg* bacteria were detected in the mouse brain, and the tightly sealed electron‐dense ultrastructure of tight junctions in the hippocampus of *Pg* group mice was indiscernible from corresponding tissues within the Con group (Figure 7A). Surprisingly, tight junctions in the hippocampus of the *Pg*‐OMV group were characterized by fainter electron density with enlarged intercellular clefts (Figure 7A). We further evaluated the protein expression of tight junctions in the hippocampus through double IF staining using occludin and the endothelial marker CD31 (Figure 7B). Compared with the Con and *Pg* groups, expression of occludin was significantly reduced in the brain endothelial cells of *Pg* OMVs group (Figure 7C). Similarly, the WB analysis also confirmed this result (Figure 7D). The protein levels of occludin in *Pg* OMVs significantly decreased by 57.625% and 54.91%, respectively, compared with those in the Con and *Pg* groups (Figure 7E).

These results suggest that *Pg* OMVs might directly disrupt tight junctions, contributing to subsequent BBB damage, which, in turn, facilitates *Pg* OMVs crossing the BBB and inducing neuroinflammation, which is different from the indirect effects of *Pg* on neuroinflammation.

## 4 Discussion

Growing evidence suggests that the oral microbiota plays an essential role in the pathogenesis of neurodegenerative diseases, including AD. *Pg*, a common oral pathogen, has been linked to an increased risk of AD [4]. OMVs derived from bacteria act as natural carriers of virulence factors that contribute to the pathogenesis of bacteria. *Pg* OMVs are reportedly involved in the development of cognitive decline in mice [17, 18]. Here, we explored the effects and underlying pathogenesis of *Pg* OMVs and *Pg* on cognitive function and neuroinflammation. We found that both *Pg* OMVs and *Pg* induced cognitive behavioral alterations and the activation of astrocytes and microglia. However, the systemic inflammatory response was triggered by *Pg*, leading to more significant activation of the NLRP3 inflammasome compared to those induced by *Pg* OMVs. While impairment of tight junction integrity was observed only in *Pg* OMV‐treated mice, indicating that there are different mechanisms by which *Pg* OMVs and *Pg* mediate neuroinflammation.

Microglia and astrocytes serve as pivotal regulators of neuroinflammation. In the normal brain, resting microglia are activated by infection, ischemic stroke, or brain trauma, which induces and enhances the inflammatory response [19]. Activated microglia express proinflammatory factors, such as IL‐1β, TNF‐α, IL‐6, and nitric oxide (NO) [33], which are secreted as inflammatory mediators to induce secondary inflammatory responses in astrocytes [34, 35]. In this study, we observed that intravenous injection of *Pg* OMVs or *Pg* increased the expression levels of IL‐1β, TNF‐α, and IL‐6; activated microglia and astrocytes in the mouse brain; and induced impaired memory behaviors. These findings suggest that *Pg* OMVs or *Pg* treatment may underlie neuroinflammation, resulting in cognitive deficits.

Although there was consistency in terms of neuroinflammation and behavioral modifications between the *Pg*‐OMV and *Pg* groups of mice, they exhibited different effects on systemic inflammation and BBB tight junctions. The spleen, as the primary sieve for invading pathogens and antigens in the bloodstream, can undergo splenomegaly in response to treatments, vascular changes, hematologic malignancies, and metabolic syndrome in mice [36]. In the present study, splenomegaly and increased proinflammatory factor levels in plasma were observed only in *Pg*‐treated mice rather than in *Pg*‐OMV‐treated mice, despite previous reports that both *Pg* and *Pg‐*OMVs can escape into the bloodstream and colonize extraoral tissues [23]. These results indicate that *Pg* and *Pg* OMV treatments induce distinct immune regulatory responses. Interestingly, *Pg* OMVs have previously been considered to promote immune evasion of *Pg* [37], although OMVs isolated from several different pathogenic microbes may exhibit alternative potent immune‐activating effects and/or promote immune tolerance to suppress inflammatory responses [38]. *Bacteroides fragilis* uses OMVs to deliver capsular polysaccharide A to dendritic cells, thereby repressing inflammation and maintaining intestinal homeostasis [39]. Accordingly, we surmise that the absence of protective assistance from OMV‐deficient *Pg* induces a marked systemic immune response resulting in splenomegaly upon entry into the circulation. Thus, *Pg* may elicit intense antimicrobial immune responses and inflammation, which induce neuroinflammation, whereas *Pg* OMVs elude the systemic immune response and inflammatory responses.

The NLRP3 inflammasome, one of the most thoroughly studied inflammasomes, plays a crucial role in the induction of neuroinflammation upon its activation [40]. We found that the expression levels of NLRP3 inflammasome‐related proteins, NLRP3, ASC, and cleaved caspase‐1, were elevated in mice treated with *Pg* and *Pg* OMVs. It is noteworthy that *Pg* induced a more pronounced activation of the NLRP3 inflammasome compared to *Pg* OMVs. This may be a result of chronic, sustained systemic, and neuroinflammatory stimulation in the mouse brain caused by *Pg*, but could also suggest other mechanisms may be responsible for *Pg* OMVs‐induced neuroinflammation.

As a highly selective permeable barrier surrounding the CNS, the BBB is the primary shield preventing pathogen entry into brain tissues [41]. However, peripheral cytokines from severe disease are suspected of inducing neuroinflammation through the BBB [42]. We found that *Pg* and *Pg* OMV treatments resulted in an increase in the number of vesicles of endothelial cells in the hippocampus and that *Pg* OMVs, but not *Pg*, induced the disruption of tight junctions between microvascular endothelial cells, which is in line with previous studies [17, 27]. As tight junctions are critical for maintaining BBB integrity and normal functioning, any changes in these junctions directly affect BBB function. However, peripheral inflammation can impair the functional integrity of the BBB without necessarily causing morphological changes [43], which may explain the lack of pronounced tight junction disruption observed in the *Pg* group. In this context, we speculate that *Pg* OMV‐induced BBB disruption, which facilitates the entry of *Pg* OMVs into the brain, is the main contributor to the observed neuroinflammation and subsequent memory defects.

LPS is the classical endotoxin of Gram‐negative bacteria. Previous studies have shown that chronic systemic exposure to *Pg*‐derived LPS in mice can activate the GSK3β/NFκB signaling pathway or induce cathepsin B to activate toll‐like receptor 2 (TLR2) signaling so as to trigger microglial‐mediated neuroinflammation. This process ultimately leads to tau hyperphosphorylation and Aβ accumulation in neurons [44, 45]. *Pg* OMVs contain many virulence factors, including LPS and various proteins. A previous study reported that mice that received an intraperitoneal injection of LPS experienced robust BBB disruption, including plasma membrane ruffling and increased brain endothelial cell transcytosis, whereas tight junction ultrastructural changes were not evident [46], which suggests that the observed damage to tight junctions might involve components other than LPS present in *Pg* OMVs. Notably, *Pg* OMVs can deliver gingipains to brain microvascular endothelial cells and decrease ZO‐1 and occludin protein levels in an in vitro BBB model [47]. Moreover, gingipains are enriched within *Pg* OMVs and, potentially, degrade tight junction proteins through protease activity, resulting in increased BBB permeability [13]. Thus, we suggest one plausible explanation for the damage to tight junctions caused by *Pg* OMVs that might occur through elevated concentrations of gingipains. Therefore, further studies investigating the effects and mechanism of *Pg* OMVs on the BBB are needed to identify specific contributors and further decipher their relationship with cognitive decline.

## 5 Conclusions

Overall, the underlying causes of neuroinflammation may differ between *Pg* cells and *Pg* OMVs. *Pg* detected by the immune system elicits a robust systemic inflammatory response. Under the sustained stimulus of chronic inflammation, this response may subsequently trigger a stronger activation of the NLRP3 inflammasome than that induced by *Pg* OMVs. Nevertheless, *Pg* OMVs initially induce BBB damage, which subsequently induces neuroinflammation and cognitive deficits. In summary, our findings not only uncover distinct pathways through which *Pg* and *Pg* OMVs contribute to disease progression but also further confirm *Pg* OMVs as important regulators of *Pg*‐induced AD‐like pathological changes.

## Author Contributions

**Yu Qiu, Guiqiong He, and Deqin Yang:** conceptualization. **Yu Qiu and Yueyang Zhao:** methodology, software, data curation, visualization, and writing–original draft preparation. **Yu Qiu, Yueyang Zhao, Guiqiong He, and Deqin Yang:** validation. **Yu Qiu and Deqin Yang:** formal analysis. **Yu Qiu, Yueyang Zhao, and Deqin Yang:** investigation. **Deqin Yang and Guiqiong He:** resources, writing–review and editing, funding acquisition, and supervision. **Yu Qiu, Guiqiong He, and Deqin Yang:** project administration.

## Ethics Statement

The animal study protocol was approved by the Ethics Committee of Chongqing Medical University (IACUC‐CQMU‐2023‐0160).

## Conflicts of Interest

The authors declare no conflicts of interest.

## Supporting information

Supporting information.

## Data Availability

All data of the present study are provided in this published article.
