# Supplementary material for: Porphyromonas gingivalis and Its Outer Membrane Vesicles Induce Neuroinflammation in Mice Through Distinct Mechanisms
Source: Immun Inflamm Dis. 2025 Feb 11;13(2):e70135. doi: 10.1002/iid3.70135 (PMC11811961; doi:10.1002/iid3.70135)
Supplement: Supplementary file 1 — Supporting information. [file IID3-13-e70135-s001.docx]

Supplementary information


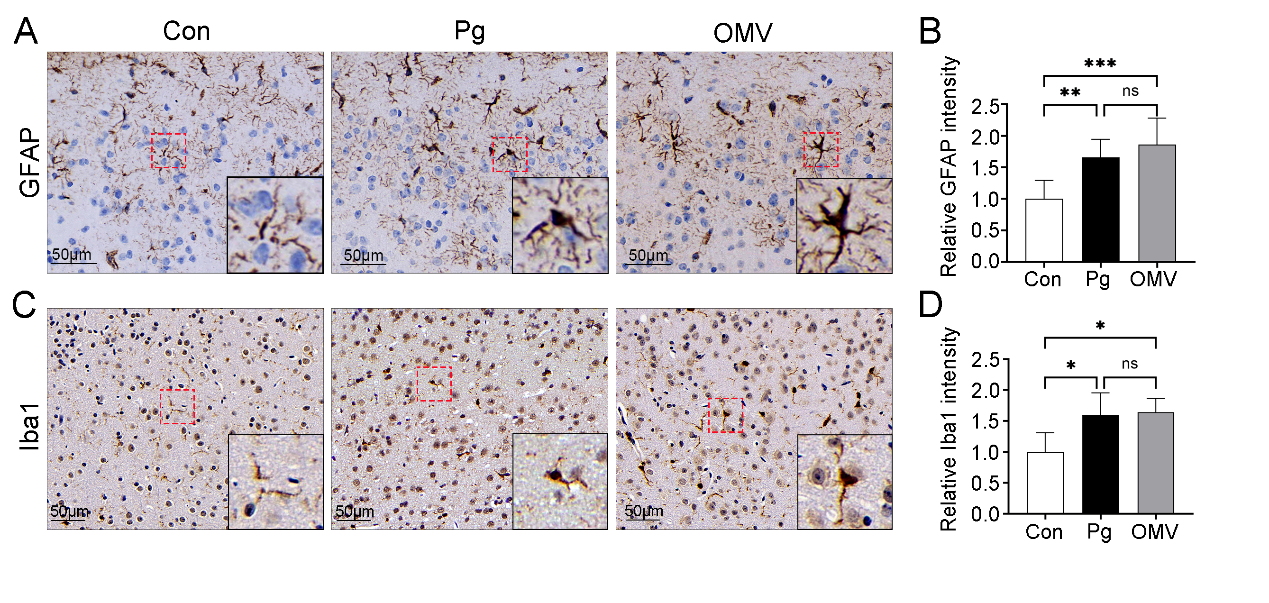


**FigureS1. *Pg* and *Pg* OMVs induced neuroinflammation in the cortex of mouse brains.** (A, C) Immunohistochemistry of GFAP and Iba-1 in cortex from the Con, *Pg* and *Pg* OMVs groups. Scale bars: 50 μm. (B, D) Quantification of the relative GFAP and Iba1 intensity in the cortex. n = 4. (E-J) IL-1β, TNF-α and IL-6 protein expression in the hippocampus. n = 3. **p* < 0.05; ***p* < 0.01; *** *p* < 0.001; ns: not significant.


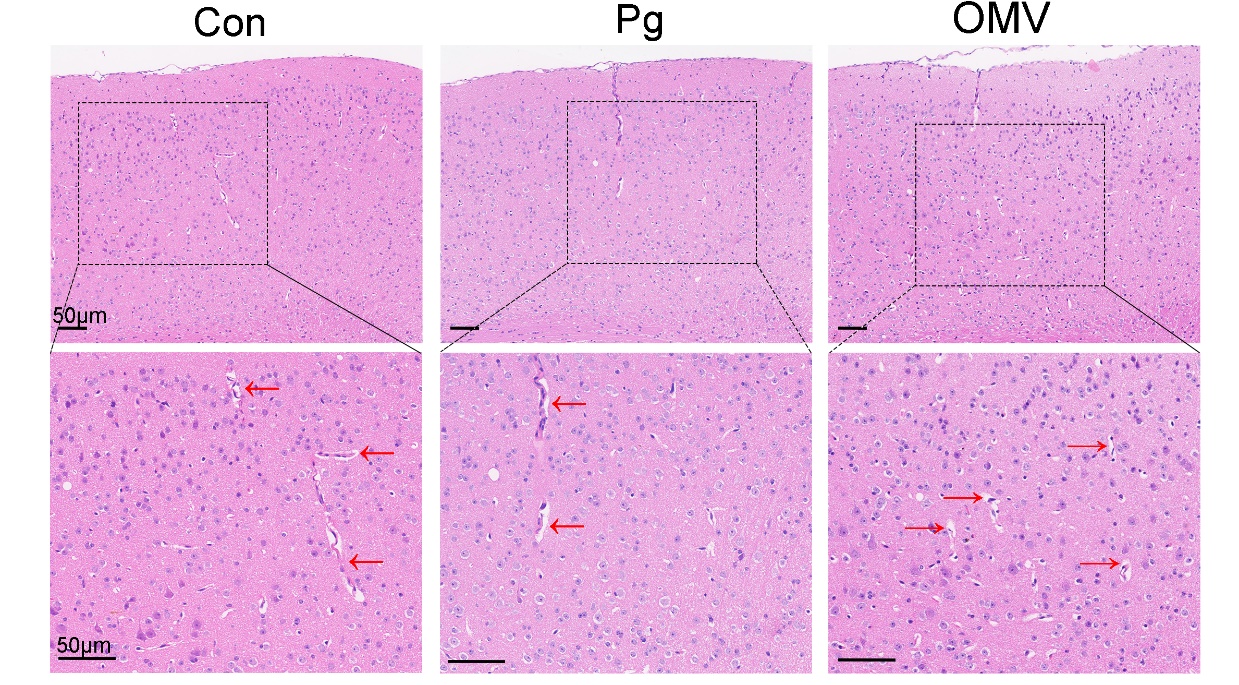


**Figure S2.** ***Pg* and *Pg* OMVs** **did not cause the vascular dilation in the cortex of mouse brains.** Representative HE-stained images of the cortex. Red arrows indicate cerebral vessels. Scale bar: 50 μm.
